# Production, Purification, and Characterization of Polygalacturonase from *Rhizomucor pusillus* Isolated from Decomposting Orange Peels

**DOI:** 10.1155/2012/138634

**Published:** 2012-10-17

**Authors:** Mohd. Asif Siddiqui, Veena Pande, Mohammad Arif

**Affiliations:** ^1^Department of Biotechnology, Kumaun University, Campus Bhimtal, Nainital 263136, India; ^2^Defence Institute of Bio-Energy Research, Nainital, Haldwani 263139, India

## Abstract

A thermophilic fungal strain producing polygalacturonase was isolated after primary screening of 40 different isolates. The fungus was identified as *Rhizomucor pusilis* by Microbial Type Culture Collection (MTCC), Chandigarh, India. An extracellular polygalacturonase (PGase) from *R. pusilis* was purified to homogeneity by two chromatographic steps using Sephadex G-200 and Sephacryl S-100. The purified enzyme was a monomer with a molecular weight of 32 kDa. The PGase was optimally active at 55°C and at pH 5.0. It was stable up to 50°C for 120 min of incubation and pH condition between 4.0 and 5.0. The stability of PGase decreases rapidly above 60°C and above pH 5.0. The apparent *K*_*m*_ and *V*_max_ values were 0.22 mg/mL and 4.34 U/mL, respectively. It was the first time that a polygalacturonase enzyme was purified in this species. It would be worthwhile to exploit this strain for polygalacturonase production. Polygalacturonase from this strain can be recommended for the commercial production because of its constitutive and less catabolically repressive nature, thermostability, wide range of pH, and lower *K*_*m*_ properties. However, scale-up studies are needed for the better output for commercial production.

## 1. Introduction

Pectin substances constitute a complex linear backbone comprised of *α*-1, 4-linked d-galacturonic acid residues which may be methylated and substituted with l-rhamnose, arabinose, galactose, and xylose [1–3]. Because of the large variety of pectins in plant material, they endowed with many pectinolytic enzyme systems which can degrade them [4]. The hydrolysis of pectin backbone is obtained by the synergistic action of several enzymes, including pectin methylesterase (EC. 3.1.11.1), endopolygalacturonase (EC. 3.2.1.15), exopolygalacturonase (EC. 3.2.1.67), pectate lyase (EC. 4.2.2.2), exo-pectate lyase (EC. 4.2.2.9), and endopectin lyase (4.2.2.10) [5, 6].

Pectinolytic enzymes are of prime importance for plants as they help in cell-wall extension and softening of some plant tissues during maturation and storage [7, 8]. They also aid in maintaining ecological balance by causing decomposition and recycling of waste plant materials. Plant pathogenicity and spoilage of fruits and vegetables by rotting are some other major manifestations of pectinolytic enzymes [9, 10]. They have been used in many industrial and biotechnological processes, such as textile and plant fiber processing, coffee and tea fermentation, oil extraction, treatment of industrial wastewater containing pertinacious material, purification of plant viruses, and paper making [11]. Commercial enzyme preparations used in processing of food, traditionally, comprising of the mixtures of polygalacturonase, pectate lyase, and pectin esterase, are almost exclusively derived from fungal species, especially *Mucor *and *Aspergillus *[12]. Preparations containing pectin-degrading enzymes used in the food industry are of fungal origin because fungi are potent producers of pectic enzymes and the optimal pH of fungal enzymes is very close to the pH of many fruit juices, which range from pH 3.0 to 5.5 [13]. Due to the potential and wide applications of pectinases, there is a need to highlight recent developments on several aspects related to their production. 

Higher cost of the production is perhaps the major constraint in commercialization of new sources of enzymes. Though, using high yielding strains, optimal fermentation conditions, and efficient enzyme recovery procedures can reduce the cost. In addition, technical constraint includes supply of cheap and pure raw materials and difficulties in achieving high operational stabilities, particularly to temperature and pH. Literature highlighting the optimization, biochemical characterization, genetics, and strain improvement studies of pectinases from mesophilic fungi is available [14–18]. However, there are few studies where stable pectinases have been reported from thermophilic fungi. Therefore in the present paper, we describe the isolation of pectinase producing fungi, purification, and characterization of polygalacturonase (PG) with appreciable activity and temperature and pH stability.

## 2. Material and Methods

### 2.1. Organism and Culture Conditions

Pectinase producing fungi were isolated from various soil, decayed fruits, and other vegetables collected from different fruits markets of western Uttar Pradesh by using modified pectin agar medium of following composition (g/L, wt/vol.) [19]: Pectin-10.0, Sucrose-10.0, Tryptone-3.0, Yeast extract-2.0, KCl-0.5, MgSO_4_·7H_2_O-0.5, MnSO_4_·5H_2_O-0.01, (NH_4_)_2_SO_4_-2.0, Agar-20.00 supplemented with mineral salt solution of composition g/100 mL CuSO_4_·5H_2_O-0.04, FeSO_4_-0.08, Na_2_MoO_4_-0.08, ZnSO_4_-0.8, Na_2_B_4_O_7_-0.004, and MnSO_4_-0.008. To the above medium distilled water was added to make 1 liter solution. The pH of the media was adjusted to 5.5–6.0 by using 1.0 N HCl/1.0 N NaOH. The salt solution with pectin, and agar were autoclaved separately. The sterilization was done at 121°C (10 lbs) for 15 minutes to avoid pectin degradation. After sterilization the pectin media and agar were mixed aseptically. Ampicillin (100 mg/mL) was also added to restrict bacterial growth. The inoculated plates were incubated at 50°C for 5–7 days. The cultures were further screened by subculturing on YPSS (Yeast soluble starch agar) medium having the following composition (g/L, wt/vol.): Pectin-15, Yeast extract-0.4, K_2_HPO_4_-0.23, KH_2_PO_4_-0.2, MgSO_4_·7H_2_O-0.05, and Citric acid-0.052. The pH of the media was adjusted to 5.5 by using 1.0 N HCl/1.0 N NaOH. Pectin utilization was detected by flooding the culture plates with freshly prepared 1% Cetrimide solution and allowed to stand for 20–30 minutes. A clear zone around the colonies against a white background (of the medium) indicates the ability of an isolate to produce pectinase. Based on screening, the isolated fungi were identified up to genus level by examining the morphological characters following Dubey and Maheshwari [20]. Highest pectinase producer isolate was selected for further studies and the culture was sent to MTCC, Chandigarh, for species identification.

### 2.2. Medium for Solid-State Fermentation (SSF) and Enzyme Production

The solid state cultivation was carried out in 250 mL Erlenmeyer flasks containing 15 g of basal medium (Pectin-0.5, Urea-0.15, Sucrose-1.57, (NH_4_)_2_SO_4_-0.68, KH_2_PO_4_-0.33, FeSO_4_-0.15, and Sugarcane bagasse-11.6). The flasks were inoculated with 2 mL spore suspension containing 10^6^ spores mL^−1^, which was obtained from a five-day agar slant. Final moisture was adjusted to 70%. The pH of the media was adjusted to 5.5–6.0 by using 1.0 N HCl/1.0 N NaOH [21]. The cultivation was carried out at 50°C for 4-5 days. The fermented media were extracted with 30 mL of distilled water. The flasks were shaken vigorously and kept for one hour and filtered through cheese cloth. The crude enzyme was extracted by adding 100 mL of citrate buffer (0.1 M, pH 5.0) to each flask. The extract was centrifuged at 10,000 rpm for 15 min at 4°C and the supernatant was filtered through Whatman No. 1 filter paper to remove spores completely. 

### 2.3. Enzyme Purification Procedure

The filtrate was centrifuged at 10,000 rpm for 20 min at refrigerated condition. Solid ammonium sulfate was slowly added to the supernatant of crude enzyme preparation so as to reach 20% saturation. Addition of ammonium sulfate was carried out with continuous stirring in an ice bath, and then it was kept at 4°C for overnight. The precipitated protein was removed by centrifugation at 10,000 rpm for 30 min at 4°C. Ammonium sulfate was added to the supernatant to 80% saturation. The precipitated protein was again separated by centrifugation at 10,000 rpm for 30 min at 4°C. The precipitated protein was dissolved in sodium acetate buffer (0.1 M; pH 5.0). The crude enzyme was loaded on a Sephadex G-200 column (1 × 50 cm) preequilibrated with sodium acetate buffer (0.1 M; pH 5.0). Fractions in 3 mL volume were collected at a flow rate of 24 mL/hour. The eluted fractions were monitored at 280 nm for protein and assayed for enzyme activity. Fraction with highest polygalacturonase activity was loaded on Sephacryl S-100 column (1.6 cm × 60 cm) preequilibrated with same at a flow rate of 20 mL/hour. Fractions of 1.5 mL were collected and monitored for proteins and polygalacturonase activity.

### 2.4. Enzyme Assay

The PGase activity was assayed by estimating the amount of reducing sugar released under assay conditions. Polygalacturonase activity was measured by determining the amount of reducing groups released according to the method described by Nelson [22] and modified by Somogyi [23]. The substrate used for assay was 1% PGA (polygalacturonic acid), that is, 1.0 g of PGA in 100 mL of 0.1 M citrate buffer, pH 5.0. The assay mixture was prepared with the following components: 0.2 mL enzyme, 0.1 mL of 0.1 M CaCl_2_, and 0.5 mL of 1% solution of polygalacturonic acid (PGA). Blank was prepared for each sample by boiling the reaction mixture before the addition of substrate. Tubes were incubated at 37°C for one hour. The reaction was stopped by heating at 100°C for 3 minutes. 0.5 mL of the solution mixture was taken and analyzed for reducing sugars by Nelson-Somogyi method. Final volume was made up to 2 mL in both sample and standard tubes with distilled water. 1.0 mL of alkaline copper tartrate was added and kept for 10 minutes. Tubes were cooled and 1.0 mL of arsenomolybdate reagent was added to each of the tubes. Final volume was made up to 10 mL volume in each tube with distilled water. The absorbance of blue color was recorded at 620 nm after 10 minutes. 

The amount of galacturonic acid released per mL per minute was calculated from standard curve of galacturonic acid. One unit of enzyme activity is defined as the enzyme that releases 1 *μ*mol mL^−1^ min^−1^ galacturonic acid under standard assay conditions.

### 2.5. Protein Determination

The protein content of the enzyme solution is estimated by the method developed by Lowry et al. [24] with Bovine serum albumin (BSA) as standard.

### 2.6. Electrophoresis

The purified enzyme was subjected to electrophoretic studies to determine molecular weight. SDS PAGE (12.5%) was conducted by using a slab gel apparatus with notched glass plate system [25]. Gels of 1.5 mm thickness was prepared using spacers of the same size. The wide range protein molecular weight marker was used for molecular weight determination of proteins. It contains myosin (205 kDa), Phosphorylase b (97.4 kDa), Bovine Serum albumin (66 kDa), Ovalbumin (43 kDa), Carbonic anhydrase (29 kDa), Soybean Trypsin Inhibitor (20.1 kDa), and Lysozyme (14.3 kDa). The gel was stained by coomassie blue prepared in 3.5% perchloric acid. After staining gel was washed and destained with acetic acid solution (7.5%) several times until band becomes visible and the background becomes clear.

### 2.7. Characterization of PGase

The substrate specificity of the purified enzyme was determined by using various substrates in the reaction mixture for enzyme assay. The various substrates used were polygalacturonic acid, pectin, xylan, galactose, and cellulose at 0.1% (w/v) [18].

The optimum temperature was estimated by performing the standard assay within the temperature range of 30–80°C. Incubating the enzyme for 4 hours at temperature from 30–80°C in assay buffer and then measuring the remaining activity by standard assay determined the inactivation temperature. The optimal pH for PGase activity was evaluated by varying the pH of the reaction mixture between 3.0 and 9.0 at increments of 1.0. Activity was then assessed under standard conditions. The effect of substrate concentration on PGase activity was assayed in standard assay by using PGA in various concentrations (0.1–1.0 mg) and *K*_*m*_ and *V*_max⁡_ were evaluated.

## 3. Results and Discussion

### 3.1. Isolation and Selection of Isolate

Thermophilic fungal strains isolated from various precollected samples from different fruits markets of western Uttar Pradesh were purified and their cultural and morphological characteristics were examined according to the method described by Dubey and Maheshwari [20]. A total of 40 fungal strains were isolated from 15 different soil, decayed fruits, and other vegetables samples. Different species of *Mucor*,* Aspergillus*, *Penicillium, Rhizopus,* and *Trichoderma* were isolated. Similar results were also reported by different coworkers: *Mucor *[26], *Aspergillus* [27], *Penicillium* [28], *Rhizopus *[29], and *Tricoderma* [30].

**Figure 1:**
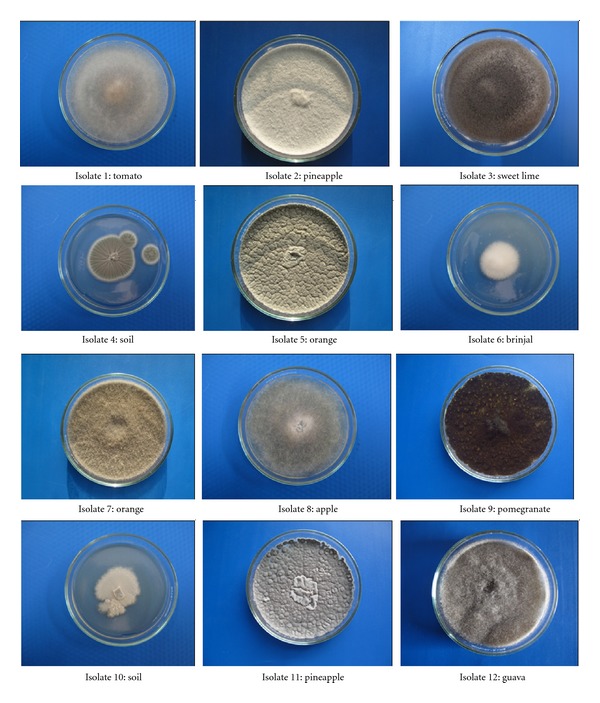
Twelve fungal isolates selected for further study on the basis of screening (5th day old culture).

High pectinase producing strains were further screened semiquantitatively by plate assay method. Twelve different isolates were further screened by solid state fermentation (Figure 1). The results indicate that isolate 7 shows maximum activity for polygalacturonase (Table 1). The isolate was further identified as *Rhizomucor pusillus* by Microbial Type Culture Collection (MTCC), Chandigarh, India.

### 3.2. Production and Detection of PGase

The production of PGase by *R. pusillus* was induced by pectin (pure) (1.5% w/v) as carbon source, urea (0.3% w/v) as nitrogen source, and MnSO_4_ (0.2% w/v) as mineral supplement at 45°C. The maximum reported polygalacturonase activity of *Mucor genevensis *is 5.0 U/mL [26], *Mucor* sp. 7 is 15.2 U/mL[31], *Penicillium viridicatum* is 18 U/mL [32], and *Mucor circinelloides *is 9.15 U/mL [18]. In the present study polygalacturonase activity was 32.57 U/mL. No report on polygalacturonase production from *Rhizomucor pusillus* is available in the literature. However it is evident from this work that this strain is a hyperproductive one and is suitable for further studies.

The crude enzyme was extracted from fermented media by a process of filtration and centrifugation to remove mycelia and other media components; the crude was concentrated to 150 mL. The crude extract contains evaluated protein and specific activity of 6.38 mg and 4.97 U/mg, respectively.

### 3.3. Purification of PGase

The purification of the PGase activity was carried out by two chromatographic steps.

First, 6.38 mg of protein from CE (150 mL) with a total activity of 31.74 U were loaded on a Sephadex G-200 column. The eluted fractions with PGase activity were identified and pooled, concentrated to 1 mL, and applied on a Sephacryl S-100 column eluted with sodium acetate buffer (0.1 M, pH 5.0). PGase activity was detected in fractions eluted between 54 and 75 mL. These last were pooled and concentrated to 1 mL.  Table 2 summarizes the steps involved in this purification as well as the specific activity, fold purification, and yield. Final specific activity of the purified PGase was 61.35 U/mg. Final fold purification and yield were 12.34 and 27.06, respectively, in standard conditions. The previous works showed a marked variation in the cases of purification factor and yield. Esquivel and Voget [33] reported more than 400 fold purification and 40% yield for *A. kawachii *IFO 4033. Mohamed et al. [34] reported 13-fold purification and 55% yield for *Tricoderma harzianum*. Celestino et al. [35] reported 9.37-fold purification and 60.62% yield for *Acrophialophora nainiana*. Saad et al. [36] reported 85-fold purification and 1.87% yield for *Mucor rouxii *NRRL 1894. Thakur et al. [18] reported 13.3-fold purification and 3.4% yield for *Mucor circinelloides. *These factors mainly depend not only on the strain but also the methods adopted for purification. Generally an increase in fold purification results in a gradual decrease in yield.

**Figure 2:**
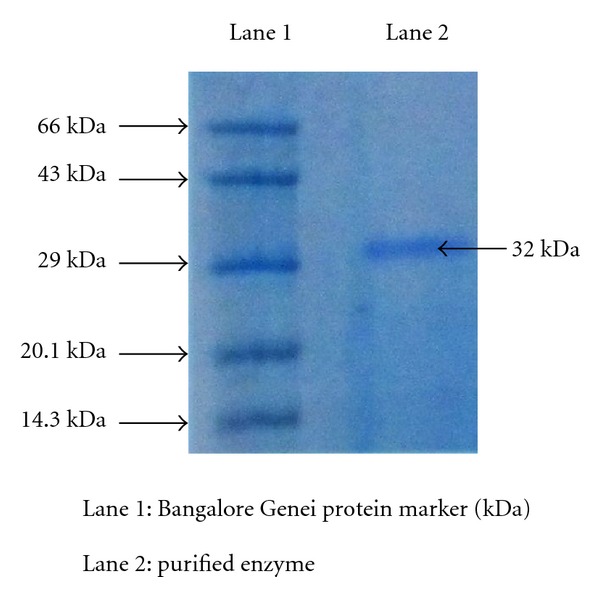
SDS PAGE of purified polygalacturonase from *Rhizomucor pusillus.* Molecular weights markers: (1) Bovine Serum Albumin—66 kDa, (2) Ovalbumin—43.0 kDa, (3) Carbonic anhydrase—29.0 kDa, (4) Soybean Trypsin Inhibitor—20.1 kDa, (5) Lysozyme—14.3 kDa.

The purified PGase was homogenous as judged by electrophoresis gel (Figure 2), where one protein band was detected after step 2. By SDS-PAGE, a molecular weight of 32 kDa was determined. Similar observations were made in *Geotrichum candidum*-34.5 kDa [37], *Rhizopus *sp. LKN-38.5 kDa [38], *Verticillium albo-atrum-*37 kDa [39], *Fusarium solani*-38 kDa [40], *Fusarium moniliforme*-30.6 kDa [41], *Trichoderma harzianum*-31 kDa [42], and *Acrophialophora nainiana-*35.5 kDa [35]. 

### 3.4. Characterization of PGase

The maximum PGases specificity was observed when polygalacturonic acid was used as substrate. Assuming it as 100%, almost half (57%) activity was expressed with pectin and only 37.8% activity was expressed with cellulose (Table 3). Previous works supporting the present study are that of Kaji and Okada [43], Kumari and Sirsi [44], Gillespie et al. [45], Shanley et al. [46], Singh and Rao [47], Mohamed et al. [34, 42], Esquivel and Voget [33], and Saad et al. [36].

**Figure 3:**
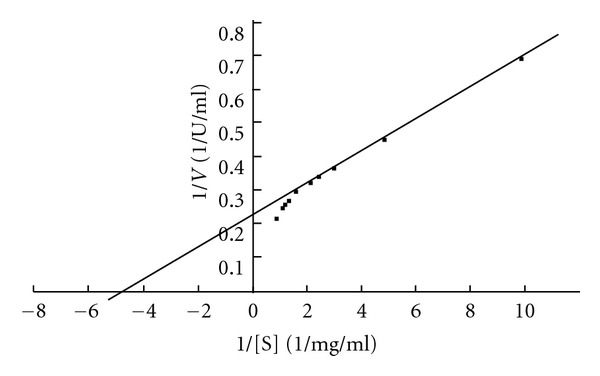
Lineweaver-Burk plot for polygalacturonase from *Rhizomucor pusillus. *

The *K*_*m*_ and *V*_max⁡_ values of PGases of *R. pusillus* are 0.22 mg/mL and 4.34 U/mL, respectively, by plotting the Lineweaver Burk plot (Figure 3). Bonnin et al. [48] reported a *K*_*m*_ of 0.071 nmol/mL and *V*_max⁡_ of 432 nkat/mg of endo-PG for *Fusarium moniliforme*. Saad et al. [36] reported a *K*_*m*_ of 1.88 mg/mL and *V*_max⁡_ of 0.045 mole/mL/min for* Mucor rouxii*. Thakur et al. [18] reported a *K*_*m*_ of 2.2 mM and *V*_max⁡_ of 4.81 IU/mL for* Mucor circinelloides*. From these studies it is evident that the kinetic properties of PG vary with the source of the enzyme. But in most cases the *K*_*m*_ value is low, which agrees with the result of the present study.

**Figure 4:**
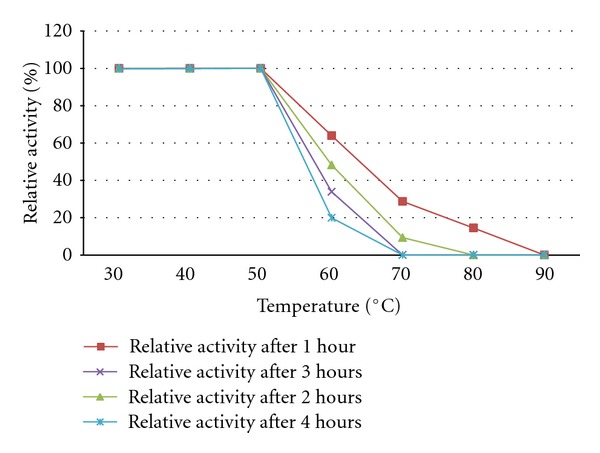
Effect of temperature on polygalacturonase stability from *Rhizomucor pusillus. *

The optimum temperature of the purified polygalacturonase from *R. pusillus* was 55°C (Table 4). The enzyme was stable at 50°C. After two hours the PG activity was only 48.2 and 9.30% at 60 and 70°C, respectively, and then the enzyme became suddenly inactive. From 80°C onwards the enzyme activity was lost during the first hour itself (Figure 4). This agreed with the earlier works of Martins et al. [49] who reported temperature optima for *Thermoascus aurantiacus *at 60°C; the enzyme was stable at 60°C for 1 hour. Kaur et al. [50] reported temperature optima for *Sporotrichum thermophile apinis* at 55°C and the enzyme was stable up to 4th hour at 65°C. Thakur et al. [18] reported temperature optima for *Mucor circinelloides* at 42°C and the enzyme was stable up to 4th hour at 42°C. Andrade et al. [51] reported the optimum temperature for polygalacturonase enzyme between 60–70°C and the enzyme retained about 82 and 63% of its activity at 60 and 70°C, respectively, after 2 hours of incubation.

**Figure 5:**
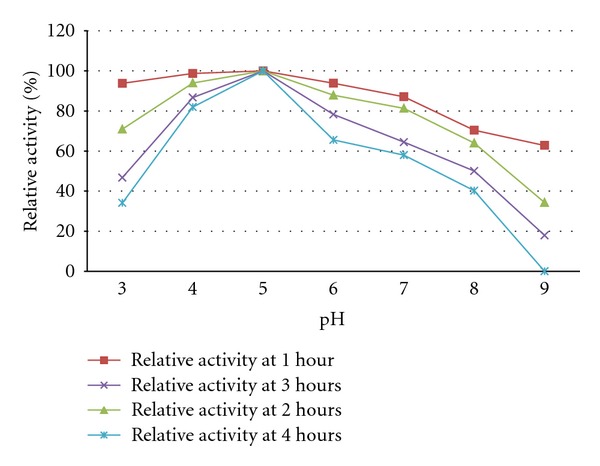
Effect of pH on polygalacturonase stability from *Rhizomucor pusillus. *

The pH optima of the purified polygalacturonase from *R. pusillus* was 5.0 (Table 5). The enzyme was stable at pH conditions 4.0-5.0. Above pH 5.0 enzyme stability began to decrease. At pH 8.0 after 4th hour the residual activity was 40.21% of that of the control. No activity was determined at pH 9.0 at 4th hour (Figure 5). Previous works supporting the present study are reported for *Aspergillussojae-*5.0 [52], *Mucor rouxii*-4.5 [36], *Mucor circinelloides*-5.5 [18], and *Cylindrocarpon destructans*-5.0 [53]. This is the typical characteristic of fungal PG [54]. In the present study it was observed that the maximum stability of the enzyme was between pH 4.0 and 5.0 followed by a fall in stability at higher pH.

## Figures and Tables

**Table 1:** Polygalacturonase activity of crude enzyme extract of different isolates.

S. no.	Isolate no.	PG activity (U/mL)
1	Isolate 1	12.78
2	Isolate 2	6.13
3	Isolate 3	19.27
4	Isolate 4	1.92
5	Isolate 5	7.81
6	Isolate 6	0.76
**7**	**Isolate 7**	**32.57**
8	Isolate 8	3.45
9	Isolate 9	9.67
10	Isolate 10	1.08
11	Isolate 11	14.10
12	Isolate 12	23.42

**Table 2:** Purification of polygalacturonase from *Rhizomucor pusilis*.

Purification step	Total activity (U/mL)	Total protein (mg)	Specific activity (U/mg)	Fold purification	Yield (%)
Crude preparation	31.74	6.38	4.97	1	100
Sephadex G-200	13.23	0.42	31.50	6.33	41.68
Sephacryl S-100	8.59	0.14	61.35	12.34	27.06

**Table 3:** Substrate specificity of polygalacturonase from *Rhizomucor pusilis*.

S. No.	Substrate (0.1%)	Enzyme activity (U/mL)	Relative activity (%)
1	Polygalacturonic acid	8.34	100
2	Pectin	4.75	57
3	Xylan	0.92	11.0
4	Galactose	0.55	6.7
5	Cellulose	3.15	37.8

**Table 4:** Effect of temperature on polygalacturonase activity from *Rhizomucor pusilis*.

S. No.	Temperature (°C)	Enzyme activity (U/mL)	Relative activity (%)
1	30	2.27	27.04
2	35	3.18	37.92
3	40	4.34	51.62
4	45	5.59	66.50
5	50	6.88	81.91
**6**	**55**	**8.41**	**100**
7	60	4.40	52.41
8	65	2.88	34.30
9	70	1.97	23.51
10	75	1.09	13.01

**Table 5:** Effect of pH on polygalacturonase activity from *Rhizomucor pusilis*.

S. No.	pH	Enzyme activity (U/mL)	Relative activity (%)
1	3	0.81	9.53
2	4	6.31	74.44
**3**	**5**	**8.47**	**100**
4	6	4.78	56.48
5	7	2.38	28.12
6	8	2.90	34.30
7	9	0.19	2.31
